# 

**Published:** 1880-01-20

**Authors:** 


### INDEX.

A

Absces's how to prevent mammary .......... 326
Abortion, hemorrhage in .......... 139
Abortions .......... 372
Abrasions of the cornea .......... 427
Acidity and pirosis, glycerine in .......... 292
Acidity as a cause of sterility .......... 373
Aconite, some points in the therapy of .......... 197
Action of sugar on calomel .......... 455
Acute rheumatism, treatment of .......... 207
Acute tonsilitis .......... 450
Adelaide Neilson, cause of death of .......... 458
Affections of the respiratory tract .......... 209
Ague, cause of .......... 69
Aihun .......... 459
Air. treatment of lung disease by condensed... 377
Albumen in urine, metaphosphoric acid a delicate test for .......... 377
Alcohol and creosote in infant diarrhœa .......... 306
Alcohol habits, erythoxylon coca on the opium and .......... 456
Alkaloid, cinchonia .......... 5
Alum mass, Bedford i on and .......... 113
Amenorrhœa from torpidity of the ovaries .......... 307
Anæsthesia with bromide of ethyl, local .......... 249
Anæsthetic, compound nitrous oxide as an .......... 62
Anæsthetics, about .......... 132
Anemia, sulphate of zinc in .......... 73
Animal electricity .......... 384
Animal substances, Wickersheimers's preservative fluid for .......... 259
Ani, pruritus .......... 181, 188
Annual deaths of the world .......... 32
Anodyne mixture .......... 267
Anthelmintic, a new .......... 340
Anthrax of fruit trees .......... 379
Anti-abortive, quinine as .......... 462
Antidote for carbolic acid .......... 187
Antidote for opium habit, a possible .......... 252
Antidotes, poisons and .......... 109
Antidote to opium, tea as an .......... 453
Anti-odontalgic .......... 75
Antiseptic, eucalyptus as an .......... 348
Antiseptic, menthol as an .......... 70
Antiseptic transfusion of human blood .......... 15
Antiseptic treatment of gun-shot wound of the knee joint .......... 274
Anus, successful treatment of imperforate .......... 258
Apparatus for the treatment of fractures of the clavicle .......... 141
Arsenic, antidote to .......... 181
Arsenic in chorea .......... 253
Arsenic in consumption .......... 462
Arsenic in neuralgia .......... 347, 428
Artificial abortion for the relief of uncontrollable nausea and vomiting, with remarks.. 275
Artificial Selter's water .......... 347
Asphyxiated, method of restoring the .......... 379
Aspiration of the bladder for retention of urine .......... 110
Asthma .......... 35, 36, 265, 302
Asthma, citrate of caffeine in .......... 138
Asthma, croup, etc .......... 115
Asthma, Dr. Bartholow's prescription for .......... 386
Atoms .......... 144, 384, 424
Atropia, action of Duboisia and .......... 97
Audiphone and dentaphone .......... 183
Audiphone, failure of the .......... 108
Auditory nerve .......... 304
Aural douche, a new .......... 396
Aural syringes .......... 239
Auscultation, telephone and microphone in... 444

B

Balm, Hagan's magnolia .......... 74
Balsam of Peru in pruritus .......... 182
Bandage after labor, should we? .......... 247
Bandage, Martin's .......... 234
Bartholow's prescription for asthma .......... 386
Battery, a new .......... 464
Bedford and alum mass .......... 113
Belladonna in obstinate constipation .......... 62
Benzoate of soda .......... 345
Benzoic acid in rheumatism .......... 341
Benzol in whooping-cough .......... 378
Beri-beri .......... 461
Bismuth, phosphate of .......... 257
Bismuth snuff .......... 179
Bites of reptiles .......... 359
Blacking, a good .......... 76
Bladder, inaction of the .......... 70
Bladder in old people, catarrh of the .......... 258
Blood of intermittents .......... 464
Blood corpuscles, wormlike derivarions from.. 263
Body, electricity in the human .......... 383
Bogus colleges finally wiped out .......... 460
Bones and sugar .......... 304
Book notices 38, 79, 119, 151, 231, 270, 311, 350, 391, 431
Boracic acid, concentrated extracts of .......... 416
Boracic acid injections in gonorrhœa .......... 421
Breathing as a pain-obtunder in minor surgery, obstetrics, etc .......... 294
Breech presentation, new method in .......... 69
Bromide of ethyl, first death from .......... 221
Bromide of ethyl, local anæsthesia wi[illegible] .......... 249
Bromidrosis of the feet .......... 420
Buboes, sulphide of calcium, in the treetment of suppurating .......... 203
Burn successfully treated by bicarbonate of soda .......... 64
Butter, rather old .......... 383

C

Caffeine in asthma, citrate of .......... 138
Calcium salicylate in the serous diarrhœa of infants .......... 387
Calculi, treatment of hepatic .......... 140
Calisaya, compound wine of iron and .......... 188
Calomel in the zymotic diseases of infancy .......... 21
Calomel, iodide of potassium and .......... 146
Calomel—its conversion into corrosive sublimate .......... 33
Cancer, clover tea for .......... 453
Cancer plaster, analysis of .......... 335
Cancer, the cure of .......... 217
Capital punishment by electricity .......... 183
Capsicum, hemorrhoids treated with .......... 262
Carbolic acid, antidote for .......... 187
Carbolic acid, compound solution of .......... 186
Carbolic acid internally in typhoid fever .......... 216
Carbolic acid, medical uses of .......... 259
Carbolic acid, systemic poisoning by the external application of .......... 379
Carbolic acid, ulceration of the bowel following the injection of a pile with .......... 459
Carbuncle, cupping in .......... 375
Carlsbad in diabetes mellitus .......... 70
Carnauba root .......... 70
Carrier pigeons at great altitudes .......... 71
Case in practice .......... 440
Cassava root, sweet .......... 180
Castor oil, to disguise .......... 345
Castration and spaying as a means of arresting syphilis—reply to Prof. Chaille .......... 41, 81
Cataract, suction operation for .......... 176
Cataract, Wolfe's operation for .......... 301
Catarrhal inflammation of the middle ear .......... 393
Catarrh, gastric .......... 73
Catarrh, nasal .......... 98
Catarrh, naso-pharyngeal .......... 131
Catarrh of the bladder in old people .......... 258
Catarrh of the bladder, lactic acid in .......... 17
Catarrh, treatment of post-nasal .......... 422
Cayaponin .......... 70

Cheloid or keloid 158
Chest diseases by petroleum, treatment of 142
Chicken cholera, Pasteur's new experim. on 201
Chloral hydrate in acute gastro-enteritis 260
Chloral hydrate, on some points in the therapy of 49
Chloral in diphtheria 73
Chloral, injury of eyes from 147
Chloral in the vomiting of pregnancy 428
Chloride of calcium for phthisis 296
Chloroform and ether 260
Chloroform, a plea for 248
Chloroform, cough mixture 308, 386
Chloroform, death from 138
Chloroform poisoning, treated with hypodermic injections of strychnia and whiskey 398
Chloroform, purity of 74
Chloroform, some remarks on 46
Chloroform vapor in earache 145
Chloroform, worms in the nasal cavities expelled by 225
Cholera and diarrhœa 307
Cholera infantum 407
Cholera infantum, lactopeptine in 268
Chorea, arsenic in 253
Chronic otorrhea, permanganate of potassa in 60
Chronic pharyngitis accidentally, yet successfully treated by a new process of cauterization 397
Cinchonia alkaloid 5, 225
Cinchona bark, the culture of cinchona tree in the United States 361
Clavicle, a simple apparatus for the treatment of fractures of 141
Climate of Georgia 78
Clinical lecture 320
Clinics, reports of 289
Clocks, self-winding 423
Coca in opium habit 420
Coffee, purgative 387
Coition, prostration and nervousness after 337
Cold as a motor 223
Cold baths in infantile convulsions 66
Cold cream without oil 33
Cold in the head 227
Colony of Victoria 72
Colloid, Richardson's styptic 227
Compound syrup of hypophosphites 425
Compressed nitrous oxide as an anæsthetic 62
Concentrated extracts, therapeutic uses of boracic acid 416
Conception on the nervous system, effects of 331
Constipation 75
Constipation, belladonna in obstinate 62
Constipation, chronic 308
Constipation, high temperature from 381
Consumption, arsenic in 461
Consumption, dyspepsia and 348
Consumption on the Salysbury plan 334
Consumption, the cure of 13
Contraction of fingers 329
Convulsions, puerperal 403
Coptis trifolia, gold thread 141
Copying ink 463
Cornea, abrasion of the 427
Corns, a remedy for 76, 115
Corns, remedy for 466
Coto bark in diarrhœa 73
Cough from elongated uvula operation 104
Cough, iodide of potassium in 262
Cough mixture 36, 308
Coughs, glycerine in 114
Coughs, wine of tar excellent for chronic 345
Coughs, oxalate of cerium in 251
Creosote in infants' diarrhœa 306
Croup, rules for the treatment of 22
Croup, tracheotomy in 103
Crushing, treatment of hemorrhoids by 413
Crusts, eczematous 377
Cubebs in whooping-cough 185
Cuticura resolvent 426

D

Damiana 147, 186
Dangers of the telephone 31
Death by drowning, signs of 97
Death from chloroform 138
Decisions, recent 100
Dentaphone, audiphone and 183
Dentistry, rapid breathing a pain obtunder in 294
Desiccated blood 466
Dextro quinine 6
Diabetes and sepsis 300
Diabetes mellitus, use of Carlsbad water in 70
Diagnosis, some cases of curious 353
Diamonds, more Scotch 224
Diamonds, novel theory about 112
Diarrhœa and dysentery, purgatives in 218
Diarrhœa, cholera and 307
Diarrhœa of infants, calcium salicylate in 387
Diathesis, prescription for the gouty 388
Digestion, iron on 341
Digitaline and atropine, action of 380
Digitalis, action and uses of 1
Diphtheria 146, 346
Diptheria, a certain remedy for 302
Diptheria, chloral in 73
Diphtheria, treatment of 468
Discutient ointment 35
Dissecting material 79
Dissemination of typhoid fever 219
Diuretics, action of various 261
Dogwood, Jamaica 251
Domestic remedy for felons 228
Douche, hot rectal 458
Dressing for superficial incised wounds 178
Duboisia and atropia, action of 97
Diphtheria 414
Diphtheria, thymic acid mixture for 267
Dynamite for stumps 261
Dysentery 226
Dysentery, enema for 307
Dysentery, veratrum in 226
Dyskinesia, uterine 375
Dysmenorrhœa 73
Dysmenorrhœa, Dr. Mussey's prescription for 146
Dysmenorrhœa, pulsatilla for 108
Dyspepsia and consumption 348
Dyspepsia in infants 465
Dyspepsia, flatulent 255
Dyspepsia, nervous debility, etc 116, 266
Dyspepsia, treatment of gaseous 468
Dyspeptic constipation 268

E

Earache, chloroform vapor in 145
Earache, alias acute catarrhal inflammation of the middle ear 393
Ear, inflammation of the middle 227
Ear, introduction of liquids in the middle 257
Eczema, oleate of lead in 227
Eczema, treatment of obstinate 113
Effects of local irritation on pain 22
Effusion, surgical treatment of pleuritic 132
Electric alarm for firemen 383
Electrical polycsope 33
Electricity, a new use for 32
Electricity, animal 384
Electricity in the human body 383
Electro-magnetic experiment 463
Elixir, Mathie's elixir 74
Elixir, simple 348
Emetic for infants 226
Enemata, nutritive 341
Engineering inventions 224
Enuresis, rhus aromatica in 233
Epistaxis and other hemorrhages, cases of 10
Epistaxis, surgical treatment of 386
Epsom salt, palatable 76
Epsom salts, disguising the taste of 468
Ergot, gangrene from the use of 410
Ergotine suppositories, treatment of fibroid tumors of the uterus by 23
Ergot in pharyngitis 266, 388
Ergot poisoning 406
Ergot, topical uses of 91 462
Erythoxylon coca on the opium and alcohol habits 456
Eserine 109
Ether and chloroform 260
Ether in sciatica, hypodermic injection of 255
Ether, spirits of nitrous 188

Ethyl, first death from bromide of........ 220
Ethyl, local anæsthesia with bromide of........ 249
Eucalyptol as an antiseptic........ 348
Eucalyptus for wounds........ 308
Eucalyptus globulus........ 299
Euonymin and iridin on man........ 178
Evidences that dead infants are born alive..... 68
Excipients........ 145
Excipient for quinia pills........ 187
Exhaustion or neurasthemia........ 385
Explosive combinations in pharmacy........ 303
Eye in connection with pregnancy, disease of 371
Eye, magnet for the removal of particles of steel and iron from the interior of the....... 261
Eyes injured by chloral........ 147
Eyes, nearsightedness and the color of the...... 30

F

Fasting or starvation........ 461
Feet, cause and treatment of bromidrosis of... 420
Feet, fetid perspiration of the........ 306
Felons, domestic remedy for........ 328
Ferri persulphatis, treatment of placenta previa by........ 108
Fever, against........ 296
Fever, case of malignant remittent........ 95
Fever, hay-........ 185, 305
Fever. hemorrhagic malarial........ 185
Fever liniment, St. Barthelemy's........ 345
Fevers, sulphite of soda in malarial........ 67
Fibroid tumors of the uterus treated by ergotine suppositories........ 23
Fibrous tumors of the uterus........ 27
Fingers, contraction of........ 329
Fish, a transparent........ 224
Fishes, voices in........ 111
Fistula, recto-vaginal........ 208
Fistula in ano, elastic ligature in........ 408
Flatulence, glycerine in........ 292
Flatulent dyspepsia and nausea in pregnancy 255
Flatulent dyspepsia, powder for........ 36
Flies from horses, how to keep........ 385
Fluids from the middle ear, for withdrawing... 453
Fluid, Wickersheimer's preservative........ 137
Fœtal monstrosities, influence of maternal shock in the production of........ 365
Food product, a plea for oleo-margarine as a... 180
Food. prolonged absence from........ 378
Forgetting personal identity........ 105
Fracture of the femoral neck, impacted........ 90
Fractures of the clavicle, simple apparatus for 141
Friction in insomnia........ 381
Fruit trees, anthrax of........ 379

G

Gall stones........ 30
Galvanic current in sciatica........ 254
Galvanic oxidation of gold........ 71
Gangrene from the use of ergot........ 410
Gastro-enteritis, chloral of hydrate in acute... 260
Gelsemium........ 291
Gelsemium in facial neuralgia........ 175
Genital organs, partial paralysis and want of co-ordination from irritation of the........ 160
Gleanings from private practice........ 315
Gleet, cure of........ 147
Glycerine in coughs........ 114
Glycerine in flatulence, acidity and pyrosis. ... 292
Glycerine in phthisis........ 142
Glycerine, tonic........ 306
Goitres, surgical treatment of........ 25
Gold thread, coptis trifoliata........ 141
Gonorrhœa, a new treatment for........ 185
Gonorrhœa, boracic acid injections in........ 421
Gonorrhœa, formula in........ 267
Gonorrhœa, mixture for acute........ 115
Gonorrhœa, non specific treatment of........ 418
Gonorrhoea, only a slight........ 467
Gouty diathesis, prescription for........ 388
Grafting, skin........ 177
Guaiacum, administration of........ 146
Guarana........ 181
Gunshot wound of the knee joint........ 274
Gyneconological facts not usually mentioned in text books........ 52
Gynecology, vaseline in........ 385

H

Hæmoptysis........ 346
Hagan's magnolia balm........ 74
Hair, falling of the........ 467
Hair, loss of........ 6
Hay asthma, turkish bath in........ 466
Hay-fever........ 185, 305
Head, for fresh cold in the........ 227
Health, influence of singing upon health........ 222
Heat, light and........ 264
Hemorrhage, arresting post partum........ 166
Hemorrhage in abortion........ 439
Hemorrhagic malarial fever........ 185
Hemorrhoids, application for painful........ 306
Hemorrhoids, crushing of........ 413
Hemorrhoids, for painful........ 4 8
Hemorrhoids, painless cure of........ 171
Hemorrhoids treated by capsicum........ 262
Hemorrhoids treated with capsicum........ 468
Hepatic calculi........ 140
Hiccough........ 107
Hodgkin's disease, description and post mortem of a case of........ 93
Hodge's pessary........ 422
Hog cholera medicine........ 147
Hoof ointment........ 346
Horses, how to keep flies from........ 385
Hot water for the induction of premature labor........ 110
How to apply the hot water vaginal douche.. 63
How to secure and hold a good practice........ 38
Human body, telephonic transmiss. through 223
Hydrastis Canadensis........ 135
Hydrate of chloral, strychnia sucesssfully antidoted by........ 339
Hydrocele, a new operation for........ 287
Hydrochlorate of ammonia, some therapeuic effects of........ 439
Hydroleine........ 314
Hyoscyamia in insanity........ 110
Hypodermic injection of ergotine in prolapsus of the rectum........ 262
Hypodermic injections of ether in sciatica, 30, 255
Hypodermic injection of morphia, unusual effect of a........ 26
Hypodermic medication........ 278
Hypodermic use of quinine........ 456
Hypodermic use, remedy for........ 108
Hypophosphites, compound syrup of........ 425
Hysterics........ 421

I

Imagination, power of........ 183
Inaction of the bladder........ 70
Incubation period of scarlatina........ 182
Indians, obstetric practices among the........ 374
Induction of premature labor by hot water..... 110
Infantile convulsions, cold bath in........ 66
Infants, an emetic for........ 226
Infants, calcium salicylate in the serous diarrhœa of........ 387
Infants, dyspepsia in........ 465
Infectious disease, tuberculosis an........ 242
Inflammation of the middle ear........ 227
Influence of tobacco smoking upon health..... 373
Influence of varicella on vaccination........ 147
Ingrowin g nails, painless operation for........ 460
Inhalation, Netolitzky's bromine........ 75
Inhalation of the nascent chloride of ammonium........ 209
Ink, copying........ 463
Insanity a symptom of uterine disease........ 164
Insanity, hyoscyamia in........ 110
Insomnia. frictions in........ 381
Intermittents. blood of........ 464
Intermittents, muriate of ammonia in........ 461
Intra-tympanic injections........ 27
Intra-uterine medication........ 107, 120
Inunctions, physiological action of fatty........ 364
Inventions, valuable sanitary........ 143
Iodide of ethyl in dyspnœa........ 297
Iodide of potassium, administration of........ 278
Iodide of potassium and calomel........ 146
Iodide of potassium in cough........ 262
Iodine a substitute for quinia........ 245

Iodine treatment of intermittent fever.............. 415
Iodoform and quinia in ulcers.......................... 147
Iodoform in otorrhœa...................................... 340
Iodoform, odorless........................................ 426
Iridin and euonymi non man.......................... 178
Iron and calisayia, compound wine of............ 188
Iron on digestion........................................... 341
Irritative cough from elongated uvula operat.. 104
Itch, aristocratic remedy for........................... 388
Itch, for barber s........................................... 348
Itching, to relieve.......................................... 306
Jaborandi in mumps....................................... 342
Jager's theory of man..................................... 424
Jamaica dogwood........................................... 251

K

Keloid or cheloid............................................ 158

L

Labor, hot water for the induction of premature........................................................ 110
Labor, should we bandage after...................... 247
Lactic acid in catarrh of the bladder................ 17
Lactopeptine in cholera infantum.................... 268
Lecture, clinical ............................................ 320
Lepra............................................................ 193
Leucocythemia.............................................. 332
Lens, a new .................................................. 364
Ligature in fistula in ano, elastic..................... 408
Light and heat................................................ 264
Lime water and milk........................... 109, 339
Liniment, St. Barthelemy's fever..................... 345
Linimentum potassii iodidi.............................. 36
Litholapaxy, case of........................................ 124
Little things in practice.................................... 79
Local irritation on pain, effects of..................... 22
Lung diseases by condensed air, treatment of 377
Lung phthisis, for night sweats in..................... 381
Lunar geology................................................ 463
Lung diseases, pneumatic apparatus in chronic............................................................ 452

M

Magnet for the removal of particles of steel and iron from the interior of the eye......... 261
Malerial fever, Prussian blue in....................... 419
Mammary abscess, how to prevent.................. 326
Martinn's bandage.......................................... 234
Materia medica, advances of........................... 212
Materia medica—Too many preparations of the cinchona bark—Culture of the cinchona tree in the United States..................... 361
Maternal shock, influence of........................... 365
Meniere's disease............................................ 18
Meniere's disease, cure of................................ 118
Menopause.................................................... 187
Menses, suppression of................................... 181
Menstruation, for irregular.............................. 185
Menstruation, prescription in disturbed........... 466
Menthol as an antiseptic.................................. 70
Metophosphoric acid....................................... 340
Metophosphoric acid a delicate test for albumen in urine............................................... 377
Metric system..................................... 20, 78 184
Microscopical examination of vaccine virus.... 451
Milk and lime water............................ 109, 339
Mines, tides in................................................ 144
Mithie's elixir.................................................. 74
Morphia, tartrate of................................ 69, 226
Motor, cold as a.............................................. 223
Move, a good................................................. 369
Mumps, jaborandi in....................................... 342
Muriate of ammonia in intermittents................ 461
Myosophobia.................................................. 133

N

Nails, painless operation for ingrowing............ 460
Nails, transverse depressions on the................ 421
Nasal catarrh.................................................. 98
Naso-pharyngeal catarrh................................. 131
Natural enemies of telegraph.......................... 72
Nausea of pregnancy....................................... 255
Nearsightedness and the color of the eyes........ 30
Needle points to the North, why...................... 463
Neonatorum, trismus....................................... 128
Nerve, auditory............................................... 304
Nervines........................................................ 387
Nervous debility, dyspepsia etc....................... 116
Nervous system, effects of conception on the... 331
Netolitzky's bromine inhalation........................ 75
Neuralgia, arsenic in.............................. 347, 428
Neuralgia cured by nerve stretching................. 129
Neuralgia, gelseminum in facial....................... 175
Neuralgia of the testis..................................... 382
Neurasthenia, or chronic nervous exhaustion 385
Neuroto-optico-ciliaris, a new operation in ophthalmic surgery........................................ 54
New battery................................................... 464
New lens........................................................ 464
Night sweats.................................................. 450
Nightsweats, for.................................... 115, 381
Nightsweats of phthisis, dover's powder for.... 182
Night temperature at different altitudes............ 31
Nitrate of amyl in uterine hemorrhage.............. 425
Nitrate of silver caustic, an improved............... 182
Nitrate of silver stains, for removing................. 262
Nitrous ether, spirits of.................................... 188
Nitrous oxide gas............................................ 61
Non-specific treatment of gonorrhœa............... 418
Nose, don't blow your nose............................. 179

O

Obstetrics, little things in................................. 20
Obstetrics, practices among the Indians........... 374
Obstetrics, rapid breathing as a pain obtunder in.................................................................. 294
Obstetrics, recent progress in.......................... 285
Oil hydrated.................................................. 313
Oil, to disguise castor..................................... 345
Ointment, hoof............................................... 346
Ointment, soothing......................................... 427
Oleate of lead in eczema................................. 227
Oleo-margarine, a plea for............................... 180
Oopherectomy................................................ 262
Operation, a daring......................................... 29
Ophthalmia neonatorum.................................. 409
Ophthalmic surgery, a new operation in............ 54
Ophthalmoscopic discovery............................. 143
Opium habit, a possible antidote for................. 252
Opium poisoning, is veratrum viride a sure antidote for.................................................... 205
Opium habit, the coca in.................................. 420
Orchitis, acute................................................ 64
Otorrhœa, iodoform in..................................... 340
Ovarian dysmenorrhœa.................................. 33
Ovaries, amenorrhœa from torpidity of the... 307
Ovariotomy.................................................... 301
Ovariotomy, parotitis as a complication of...... 460
Ovariotomy, when to perform........................... 218
Oxide, remakable behavior of silver................. 144
Ozena........................................................... 221
Ozena, recent suggestion for........................... 116

P

Painless cure of internal hemorrhoids.............. 171
Painless operation for ingrowing nails.............. 460
Pains, for after-............................................... 188
Paper pulp from poplar wood.......................... 143
Paralysis and want of co-ordination from irritation of the genital organs......................... 160
Paralysis, is it inherited................................... 160
Paraphimosis, simple mode of reducing a...... 227
Parotitis as a complication of ovariotomy...... 460
Parvules........................................................ 113
Pelvic hematocele........................................... 448
Peripheral paralysis of the face, new mode of distinguishing central from............................ 342
Permanganate of potassa in chronic otorrhœa 60
Pertusis, a pad for.......................................... 383
Petroleum, treatment of chest disease by......... 142
Pessaries, use of............................................. 360
Pessary, Hodge.............................................. 422
Pharmacy, explosive combinations in.............. 308
Pharyngeal catarrh.......................................... 131
Pharyngitis, ergot in............................... 266, 388
Phosphate of bismuth...................................... 257
Phosphorus and its combinations..................... 142
Photography under water................................ 344
Phthisis, chloride of calcium for....................... 296
Phthisis, counter-irritation in febrile.................. 142
Phthisis, for................................................... 348

Phthisis, glycerine in.... 142
Picrotoxin in night sweats.... 308
Piles, for external.... 305
Piles, purgative in.... 76
Pilocarpin in the treatment of malarial fever 441
Placenta prævia, method used in Germany in the treatment of.... 28
Placenta prævia, treated by ferri persulphatis 108
Plaster, analysis of a secret cancer.... 355
Pleuritic effusions, surgical treatment of.... 132
Ploughs, the king of.... 112
Pneumatic apparatus in chronic lung diseases 452
Pneumonitis.... 174
Podophylin.... 380
Poisoning, a remarkable case of.... 358
Poisoning by rhus toxicodendron.... 308
Poisoning by strychnia.... 386
Poisoning by the external application of carbolic acid.... 379
Poisoning, ergot.... 406
Poisoning, ergot in.... 266
Poisoning, treatment of strychnia.... 65
Poisonous vinegar.... 69
Poisons and antidotes.... 109
Polyscope.... 304
Popular nostrums.... 25
Post-mortem of a case of Hodkin's disease.... 93
Postpartum hemorrhage, new method of arresting.... 166
Potash, salicylate of.... 265
Potassium and calomel, iodide of.... 146
Poultice, how to make.... 426
Power of imagination.... 183
Pregnancy, disease of the eye occurring in connection with.... 371
Pregnancy, nausea in.... 255
Pregnancy, the vomiting of.... 305
Pregnancy, to arrest vomiting during.... 36
Pregnant and puerperal woman, rubeola in a.. 180
Preservative fluid.... 467
Prolapse of the ovaries.... 448
Prolapsus of the rectum treated by hypodermic injections of ergotine.... 362
Prolapsus uteri, new operation for.... 261
Prophylactic, water as a.... 368
Propylamine in acute rheumatism.... 30
Prostatic enlargement.... 33
Prostration and nervousness after coition.... 337
Pruritus ani.... 181, 188
Pruritus ani, balsam of Peru in.... 182
Pruritus vulvæ.... 465
Pterygium, operation for.... 109
Puerperal convulsions, death from.... 403
Puerperal fever, treatment of.... 462
Pulsatilla for dysmenorrhœa.... 108
Purgative coffee.... 387
Purgatives in dysentery and diarrhœa.... 218
Purgatives, tasteless saline.... 33
Purpurea, remedies for.... 307
Pyrogallic acid in syphilitic ulcerations.... 222
Pyrosis, glycerine in.... 292

Q

Quarantine by the general government, reply to Prof. L. S. Joyne's papers on.... 41, 81
Quinia and iron tonic.... 34
Quinia in ulcers.... 147
Quinia, iodine a substitute for.... 245
Quinia pills, excipient for.... 187
Quinine, action of.... 380
Quinine as an anti-abortive.... 462
Quinine, a substitute for.... 342
Quinine, hypodermic use of.... 456
Quinine in connection with sedatives.... 336
Quinine in whooping cough.... 116
Quinine, therapeutic action of.... 113

R

Rectal douche, hot.... 458
Recto-vaginal fistula cured without operation 208
Rectum, specular examination of.... 376
Reply to Prof. Levin S. Joyne's paper on "Quarantine by the Government," and to Prof. Chaille on "Castration and Spaying as a means of arresting syphilis.... 41, 81
Reports of clinics.... 289
Reptiles, bites of.... 359
Resection of the infra-orbital nerve for the cure of a tic douloureux of twelve months standing.... 438
Respiratory tract, active and passive inhalation of chloride of ammonium in acute affections of the.... 209
Restoration after the hand is completely separated from the arm.... 327
Resuscitation of a still-born infant nearly an hour after birth.... 175
Rheumatic origin, sciatica of.... 338
Rheumatism.... 267
Rheumatism, benzoic acid in.... 341
Rheumatism, cure of.... 34
Rheumatism, for acute.... 76
Rheumatism, prophylamine in acute.... 30
Rheumatism, salicin and salicylic acid in acute.... 101
Rheumatism, sequelæ of.... 86
Rheumatism, senecio aureus in.... 187
Rheumatism, treatment of.... 207
Rhinoplastic operation, a new.... 67
Rhus aromatica in enuresis.... 233
Rhus toxicodendron, poisoning by.... 268, 308
Richardson's styptic colloid.... 227
Right-handed, why are we.... 343
Ringworm of the scalp.... 170
Rubeola in a pregnant and puerperal woman.... 180
Rules for the treatment of croup.... 22

S

Saint Barthelemy's fever liniment.... 345
Salicin and salicylic acid in acute rheumatism 101
Salicylate in the serous diarrhœa of infants.... 387
Salicylate of potash.... 265
Salicylic acid, a palatable solution of.... 347
Saline purgatives, tasteless.... 33
Salisbury plan, consumption on the.... 234
Sanitary inventions, valuable.... 143
Sanitas.... 224
Scalp, ringworm of the.... 170
Scarlatina, cold wet sheet pack in.... 200
Scarlatina, incubation period of.... 182
Scarlet fever, warm baths in.... 457
Sciatica, galvanic current in the treatment of 254
Sciatica, hypodermic injections of ether... 30, 255
Sciatica of rheumatic origin.... 338
Scioric acid.... 70
Sedatives, the use of quinine in connection with.... 336
Selter's water, artificial.... 347
Senecio aureus in rheumatism.... 187
Sepsis, diabetes and.... 300
Sequelæ of rheumatism.... 86
Sex at will, production of.... 323
Silver oxide, remarkable behavior of.... 144
Simple elixir.... 348
Sun's rays as a means of research.... 223
Singing upon health, influence of.... 222
Soda, benzoate.... 74, 345
Solution of carbolic acid, compound.... 186
Skin diseases, sulphur in.... 250
Skin grafting.... 77
Snakes, about.... 111
Snakes, bites.... 274
Sneezing, how to cure fits of.... 68
Snuff, bismuth.... 179
Sore nipples, formula for.... 467
Spasm, wind.... 136
Special notices 40, 80, 120, 152, 192, 232, 252, 27, 272, 312, 352, 392
Spectacles, how to postpone the use of.... 140
Specular examination of the rectum.... 376
Speculum always at hand, Sim's.... 169
Spermatoza, structure of.... 424
Spirits of nitrous ether.... 188
Spiritualism and science.... 423
Stains, for removing nitrate of silver.... 262
Stammering, treatment of.... 343
Starvation or fasting.... 461
Sterility, acidity as a cause of.... 373
Strychnia and whiskey in chloroform poison-hypodermic injections of.... 398
Strychnia poisoining, treatment of.... 65, 386
Strychnia, successfully antidoted by hydrate of chloral.... 339

Stumps, dynamite for 263
Stypic colloid, Richardson's 227
Submarine telephone 112
Suction operation for catarrh 176
Sugar, bones and 304
Sugar on calomel, action of 455
Sulphate of zinc in anemia 73
Sulphide of calcium in the treatment of suppurating buboes 203
Sulphite of soda in malarial fevers 67
Summer complaint 462
Supra-orbital neuralgia, cured by nerve stretching 129
Surgical treatment of epistaxis 386
Syphilis, for 35
Syphilis in relation to marriage 417
Syphilitic neuralgia, iodoform in 467
Syphilitic ulcerations treated by pyrogallic acid 222
Syringes, aural 237
Systemic poisoning by the external application of carbolic acid 379

T

Tan and freckles, for the removal of 348
Tanner's fast 310, 330
Tarsal ophthalmia 177
Tartrate of morphia 226
Tea as an antidote to opium 453
Tea for cancer, clover 453
Tea, toxical effects of 298
Telegraph, natural enemies of 72
Telegraph, seeing by 264
Telephone 344
Telephone and microphone in auscultation 444
Telephone, dangers of the 31
Telephone in Scotland 223
Telephone, submarine 112
Telephonic transmission through the human body 223
Temperature from constipation, high 381
Testis, neuralgia of the 382
Therapeutic action of quinine 113
Therapeutic effects of hydrochlorate of ammonia 439
Therapeutic uses of boracic acid 416
Therapy of aconite 197
Thomas' scoop or spoon, removal of uterine fibroid with 370
Thymic acid mixture for diphtheria 267
Tic douloureux of twelve months standing cured by resection of the infra-orbital nerve and its branches 438
Tides in mines 144
Time saving 342
Tonga 299
Tongue 220
Tonic glycerine 306
Tonic wine 265
Tonsilitis, acute 450
Tonsils, treatment of diseases of 215
Tooth brushes 29
Topophone 184
Torpidity of the ovaries 307
Toy, new philosophical 32
Tracheotomy in croup 103
Tracheotomy, substitute for 381
Transplantation of skin from the sheep to the human body 414
Trismus neonatorum 128
Traumatic tetanus 28, 126
Tubercular laryngitis, for the cure of 468
Tuberculosis an infectious disease 242
Tumors connected with the uterus 256
Typhoid fever 433
Typhoid fever as treated in the Philadelphia hospital 399
Typhoid fever cured by carbolic acid internally 216
Typhoid fever, dissemination of 219
Typhoid fever, etiology of 118
Typhoid fever, prevention of the spread of 411

U

Ulceration of the bowel following the injection of a pile with carbolic acid 459
Ulcerations, syphilitic 222
Ulcers, iodoform and quinia in 147
Urethrotomy by Wheelhouse's method 58
Urinary organs, points in surgery of 454
Urine, aspiration of the bladder for the retention of 110
Urine, metaphosphoric acid a delicate test for albumen in 377
Uterine disease, insanity a symptom of 164
Uterine dyskinesia 375
Uterine fibroid removed with Thomas' scoop 370
Uterine hemorrhage, nitrate of amylin in 425
Uterine medication 107, 120, 237
Uterus, removal of the 256
Uterus, treatment of the fibrous tumors of the 27

V

Vaccine virus, microscopic examination of 451
Vaginal douche, how to apply hot water for 63
Vapor in earache, chloroform 145
Varicella on vaccination, influence of 147
Varnish, Chinese 468
Venesection 179
Veratrum viride 283
Veratrum viride a sure antidote for opium poisoning in the acute form; may it not be in its chronic, opium inebriety? 205
Veratrum viride in dysentery 226
Vermifuge 226
Vinegar, poisonous 69
Voice in fishes 111
Vomiting during pregnancy 305
Vomiting during pregnancy, to arrest 36
Vomiting of pregnancy, chloral in 428

W

Warm baths in scarlet fever 457
Warts, venereal 428
Was the child born premature or at full term? 65
Watch dog, a mechanical 184
Water as a prophylactic and a remedy 368
Water-proof varnish 428
Wet sheet pack in scarlatina, cold 222
Wheelhouse's method in extern. urethrotomy 58
Whooping cough, benzol in 378
Whooping cough, cubebs in 185
Whooping cough mixture 34
Wickersheimer's preservative fluid 137, 259
Wind spasms 136
Wine of tar excellent for chronic coughs 345
Wine, tonic 265
Winking portrait 344
Winter of 1879-80 184
Woolfe's method for cataract 301
Wormlike derivations from blood corpuscles 263
Wound dressing 428
Wounds, eucalyptus for 308
Wounds, new dressing for superficial incised 178

Y

Yellow fever, a more indigenous than exotic disease, and not belonging to the zymotic in the shape of contagion 195
Yellow fever germ, no 380
Yellow fever, some objections to the present theory regarding 131
Yellow fever, treatment of 130

Z

Zymotic diseases of infancy, value of calomel in 21

### INDEX OF AUTHORS.

BARTOW, BERNARD.—A new operation for the radical cure of hydrocele.......... 287
BLAKE, C J.—The telephone and audiphone in auscultation.......... 444
BOYD, S. S.—How to prevent mammary abscess.......... 326
BRANNON, T. C.—Domestic remedy for felons 328
BROWN, IRA.—Recto-vaginal fistula, cured without operation.......... 208
COMPTON, J. W.—The therapeutic action of quinine.......... 113
CULVER, DUDLEY M.—Cold wet sheet pack in scarlatina.......... 200
Cholera infantum.......... 407
DAWSON, W. W.—Martin's bandage.......... 234
EGAN, J. H.—Hydroleine—hydrated oil.......... 313
Typhoid fever.......... 434
ELWEL, ALEXANDER.—Should we bandage after labor and why? And, if not, why not? 247
GOODELL, WM.—Prolapse of the ovaries—Pelvic hematocele.......... 448
GREENLEY, T. B.—Cinchonia alkaloid.......... 5
Dextro-quinine.......... 6
Some objections to the present theories regarding yellow fever.......... 153
GRINNELL, FORDYCE.—Iodine a substitute for quinia.......... 245
HALDEMAN, J. S.—If veratrum viride is a sure antidote for opium poisoning in the acute form, may it not be in its chronic opium inebriety? An affirmative opinion 205
HARRISON, W. K.—The physiological action of fatty inunctions.......... 364
HAZLEWOOD, A.—Some remarks on chloroform.......... 46
HENDREE, J.—Bites of reptiles.......... 358
A case in practice.......... 440
HERRICK, O. E.—Four gynecological facts not usually mentioned in text books.......... 52
HICKMAN, J. W.—On some points in the therapy of chloral.......... 49
On some points in the therapy of aconite 197
HOBBS, A. G.—The action and uses of digitalis.......... 1
Sequelæ of rheumatism.......... 86
HOLLOWAY, J. A.—Antiseptic treatment of gunshot wound of the knee joint.......... 274
HOUSTON, THOMAS F.—Common earache, *alias* acute catarrhal inflammation of the middle ear.......... 390
JENKINS, J. T.—Cure of Meniere's disease.......... 118
Tic douloureux of twelve months standding cured by resection of the infra-orbital nerve and its branches.......... 438
JOHNSON, LINDSAY.—Keloid or cheloid.......... 158
JONES, JOSEPH.—Snake bites.......... 274
JORDAN, M. H.—A case of artificial abortion for relief of nausea and vomiting, with remarks.......... 275
KEATING, JOHN M.—Ergot poisoning.......... 406
KERSTAN, F. F.—A remarkable case of poisoning.......... 358
KINNE, A. F.—A case of impacted fracture of the femoral neck.......... 90
Some cases of curious diagnosis.......... 353
LALLERSTEDT, J. L.—Rhus aromatica in enuresis.......... 233
LARUE, J. ALEXANDER.—Treatment of acute rheumatism.......... 207
LOW, JAS. H.—Lepra.......... 193
LUNDY, C. J.—Neurotomy optico-ciliaris—a new operation in ophthalmic surgery.......... 54
MACEWEN, WILLIAM.—Antiseptic transfusion of human blood.......... 15
MAKENZIE, D. L.—Case of remittent malignant fever.......... 95
MITCHELL, THOMAS S.—Some of the therapeutic effects of hydrochlorate of amm.......... 439
NICOLSON, W. PERRIN.—Case of chloroform poisoning, treated with hypodermic injections of strychnia and whiskey.......... 398
NORWOOD W.C.—Veratrum viride.......... 283
OTIS, FESSENDEN N.—Sulphide of calcium in the treatment of suppurating buboes... 203
PALMER, C. D.—Intra-uterine medication.......... 120
PAYNE, ALBAN S.—Reply to Prof. Levin S. Joynes' paper on "Quarantine by the Government," and to Prof. Chaille on "Castration and Spaying as a means for arresting Syphilis.......... 41, 81
PROUT, J. S.—Aural syringes.......... 239
RICHARDSON, W. L.—Recent progress in obstetrics.......... 185
SANGER, A. F.—A case of litholapaxy.......... 124
SAYRE, LEWIS.—Partial paralysis and want of co-ordination from irritation of the genital organs.......... 160
SHOEMAKER, JOHN V.—Loss of hair.......... 6
SMYTHE, A. G.—Too much materia medica—Too many preparations of the cinchona bark—The culture of the cinchona tree in the United States.......... 361
STATON, L. L.—Restoration after the hand is completely separated from the arm.......... 327
STIDHAM, J. S.—Chronic pharyngitis accidentally, yet successfully treated by a new process of cauterization.......... 397
SUGGS, I. T.—Use of pessaries, etc.......... 360
TYNER, T. J.—Hypodermic medication.......... 278
UPSON, C. R.—A new aural douche.......... 396
VAN BUREN, H.—Traumatic tetanus.......... 126
VANDERBECK, C. C.—Gleaning from private practice.......... 315
WEATHERS, L. W.—Is it inherited paralysis 160
WEBER, S. G.—Water as a prophylactic and a remedy.......... 368
WILSON, H AUGUSTUS.—Clinical lecture... 320
WILSON, THOMAS.—On the influence of maternal shock in the production of fœtal monstrosities.......... 365
WINSLOW, RANDOLPH.—Some cases of epistaxis and other hemorrhages.......... 10

### EDITORIAL AND MISCELLANEOUS.

*Contributors to last Volume.*—By an oversight in our index of last year's volume the contributors to our *Practical Notes* department were omitted. It was unintentional and resulted from the great press of business upon us.

We here append the names of the medical gentlemen who favored us with articles to this department, and request that they continue to write, and that others favor us with notes of practice and useful formulae during the present year :

W. D. Hunt, M.D., of Kentucky.
Jas. Case, M.D., of Georgia.
I. T. Suggs, M.D., of Texas.
J. C. McCoy, M.D., of Texas.
W. W. Carpenter, M.D., of California.
R. E. Hutchins, M.D., of Mississippi.
McCready, M.D., of Pennsylvania.
A. W. Burrows, M.D., of Indiana.
L. A. Rutherford, M.D., of Georgia.
L. G. Lincecum, M.D., of Texas.
J. S. Jones, M.D., of Louisiana.

*Medical Societies.*—We again urge medical men in every community to organize Medical Societies. They constitute an excellent means of promoting progress in the profession. They bring medical brethren into close and intimate relations, and develop friendly and social qualities among them ; they impart zeal and interest to the study of medical science, and furnish the opportunity for pleasant and instructive interchange of views upon medical topics, which redounds to the mutual benefit of the members.

We have offered and still offer special concession to Medical Societies in ordering of our journal.

### *EDITORIAL NOTICES.*

*Greetings from Advertising Patrons.*—We are pleased to acknowledge New Year greetings from our esteemed advertising patrons, Messrs. Parke, Davis & Co., and Messrs. Hance Brothers & White. We tender them our thanks and wish them great happiness and abundant success.

*Henry Thayer & Co.*—See new advertisement of this old and reliable establishment, commencing with this number of our journal. The purity and excellence of their resinoids and sugar-coated pills are well attested. They present a fine list of *New Remedies*, and their specialties are of a high order.

*Parke, Davis & Co.*—We ask attention to the new advertisement of this large, reliable and enterprising House, commencing with this issue of our journal.

*Wm. R. Warner & Co's new Advertisement.*—This excellent House maintains unabated the confidence of the public .Their business is very extensive.

*How to Secure and Hold a Good Practice*.—Be attentive and courteous to all. Go promptiy to any call. Show an interest in your patient; if you do not feel, then assume it; it need not be considered deception, but is truly and essentially a good medicine. It helps the patient. Do not be over-hasty, but as far as possible use dispatch and give an appearance of decision and confidence. Stay at your office or place of business when not engaged in practice; and when gone leave a record on a slate, stating when you will return. In addition to this, keep well posted in the progress of the profession, that you may give your patients the benefit, and yourself the advantage, of the latest and best facilities. To do this you should take THE SOUTHERN MEDICAL RECORD.

*Scott & Browne's Preparations of Cod Liver and Palatable Castor Oil*, advertised in this journal, are both excellent medicines, in which two very useful and important medicinal agents are presented to the profession in neat and palatable form. See advertisement.

*Atlanta Price Current of Drugs*.—The price current of drugs in this city will be hereafter published regularly in our journal—corrected monthly by the wholesale and reliable drug House of Messrs. Pemberton, Pullum & Co. This will add to the interest and usefulness of THE RECORD, and will, we doubt not, be appreciated by our readers.

*Our Advertising Department* costs our subscribers nothing, as we give a uniform amount of reading matter with each issue—it is growing in interest and variety—we hope that our readers will patronize our advertisers. As a rule, men who thus announce their business to the world, are prompt, energetic and liberal men, and deserve to be sustained. We feel safe in recommending all who are represented in our pages as *first-class and reliable houses*.

---

### *BOOK NOTICES.*

### *RECEIPTED.*

[Receipts not acknowledged privately are entered here.]

1880: Arnold & Quinn; E. Y. Flemming; T. S. Jones; P. W. Calleham; John Gerdine; T. B. Miller; R. Blacknall; W. B. King; T. P. Oliver; I. E. Terrell; A. P. Harris; W. I. Kindall; Harrison; W. S. Beale; R. T. Edwards; E. B. Young; E. R. Willis; A. B. McWhorter; W. S. Glass; J. W. Unger; T. J. Jones; E. K. Bozeman; H. L. Sutherland; K. H. Davis; E T. Hunt; M. E. [illegible]emaret; A. Atkinson; W. H. Green; R. T. Matthews; C. M. Gibson; J. M. Stansill; J. M. Covington; H. M. Salley: E. V. Wheeler; T. M. Palmer; I. H. Boggan. 1879. W. Sellers; D. C. Readle.

---

